# Targeting EBV Episome for Anti-Cancer Therapy: Emerging Strategies and Challenges

**DOI:** 10.3390/v17010110

**Published:** 2025-01-15

**Authors:** Febri Gunawan Sugiokto, Renfeng Li

**Affiliations:** 1 Program in Microbiology and Immunology, University of Pittsburgh, Pittsburgh, PA 15219, USA; fgs15@pitt.edu; 2 Cancer Virology Program, Hillman Cancer Center, University of Pittsburgh Medical Center, Pittsburgh, PA 15232, USA; 3 Department of Microbiology and Molecular Genetics, University of Pittsburgh, Pittsburgh, PA 15219, USA

**Keywords:** Epstein–Barr virus, cancer, episome, reactivation, anti-cancer therapy, CRISPR

## Abstract

As a ubiquitous human pathogen, the Epstein–Barr virus (EBV) has established lifelong persistent infection in about 95% of the adult population. The EBV infection is associated with approximately 200,000 human cancer cases and 140,000 deaths per year. The presence of EBV in tumor cells provides a unique advantage in targeting the viral genome (also known as episome), to develop anti-cancer therapeutics. In this review, we summarize current strategies targeting the viral episome in cancer cells. We also highlight emerging technologies, such as clustered regularly interspersed short palindromic repeat (CRISPR)-based gene editing or activation, which offer promising avenues for selective targeting of the EBV episome for anti-cancer therapy. We discuss the challenges, limitations, and future perspectives associated with these strategies, including potential off-target effects, anti-cancer efficacy and safety.

## 1. Introduction

The Epstein–Barr virus (EBV) was discovered from Burkitt lymphoma cells in 1964 [1], and it is estimated that over 90% of the adult population worldwide has been infected with EBV at some point in their lives, mostly asymptomatic. However, in sub-Saharan Africa, where *Plasmodium falciparum* malaria is highly prevalent, EBV infection may contribute to the development of Burkitt lymphomas (BLs) in children, commonly referred to as endemic BLs [2]. In other places, EBV infection is detected in up to 30% of BL cases [3]. EBV infection in adolescents and young adults can lead to mononucleosis [4], which is associated with an increased risk of developing EBV^+^ Hodgkin Lymphoma (HL) [5]. In addition to lymphomas in immunocompromised patients (such as HIV-associated and post-transplant cases), EBV infection is associated with most cases of nasopharyngeal carcinoma (NPC) in southern China, southeast Asia, and north Africa, though genetics and environmental factors also contribute to its development [6,7,8]. It is estimated that 10% of gastric cancer cases are linked to EBV infection, referred to as EBV-associated gastric cancer (EBVaGC) [9,10,11]. Virus latent gene expression is considered to be a driver of the oncogenesis in EBV-positive malignancies, but increasing evidence suggests that lytic infection also contributes to the early stages of EBV-induced tumor development [12,13]. Furthermore, EBV infection is associated with multiple sclerosis, a neurodegenerative disease that affects the central nervous system [14,15,16,17].

EBV is primarily transmitted through oral routes, where it initially targets epithelial cells for lytic infection. During this phase, the virus actively replicates and produces new virions. Subsequently, EBV infects B cells to establish a latent state, during which the viral genome becomes circularized and chromatinized as an episome, resembling the tightly packed form of cellular chromatin, which contributes to the repression of lytic genes [18]. Periodically, EBV reactivates from latency to produce new virions, which enable the virus to infect other B cells and epithelial cells, facilitating its spread within the host and transmission to other individuals. Although significant progress has been made toward our understanding of EBV infection, replication, and viral pathogenesis, there are no viral-specific therapeutic strategies to treat EBV-associated cancer. The development of viral-specific therapeutics is hindered by the lack of suitable virus-specific targets in cancer cells, where EBV stays latent and is expressed in only a limited set of genes. Another challenge stems from the potential off-target effects of targeting host proteins. Furthermore, the development of EBV-specific therapies is restricted by the lack of robust animal models that accurately mimic EBV infection and associated cancers in humans.

EBV typically persists with over 10 episomes per malignant or transformed cell, with copy numbers well-characterized in various cell lines. Akata BL cells contain approximately 20 copies of the EBV genome per cell [19], while Mutu cells harbor 30–45 copies [20]. The B95-8 marmoset lymphoblastoid cells carry about 18 copies per cell [21]. Raji cells, derived from a Nigerian BL patient and characterized by defective virus production, contain approximately 55 copies per cell [22]. A population study reported EBV copy numbers ranging from 16 to 29 across 915 lymphoblastoid cell lines (LCLs) from the HapMap and 1000 Genomes projects [23]. In EBVaGC cells, EBV can maintain up to 800 episomes per cell [24]. The unique presence of EBV episomes in tumor cells provides a way to specifically target EBV for anti-cancer therapy. In this review, we will discuss the current research status of targeting viral episome as anti-cancer strategies.

## 2. Targeting the EBV Episome for Anti-Cancer Therapy

### 2.1. Targeting EBV Episome Maintenance Protein EBNA1

**Figure 1:**
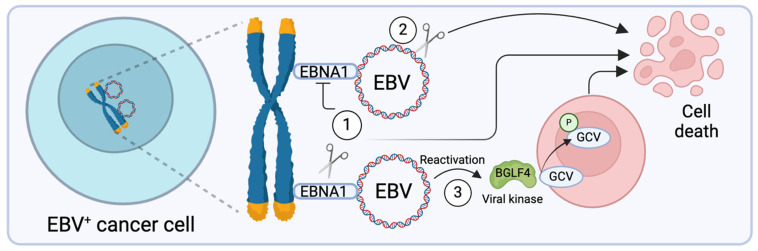
In latently infected cells, EBV episomes are tethered to host chromosomes by EBNA1. There are three major approaches to eliminating EBV-positive cancer cells: (1). EBNA1 Inhibition/Degradation: EBNA1 tethers the EBV episomes to the host chromosomes, and targeting this protein can lead to episome loss and cell death. (2). Direct Genome Cleavage: This strategy involves using tools like CRISPR/Cas9 to cut and remove the EBV genome. (3). Lytic Reactivation Therapy: This involves reactivating the virus into its lytic phase, allowing antiviral drugs [e.g., ganciclovir (GCV)] to selectively target and kill EBV-infected cells through the activity of viral protein kinase BGLF4. (Created with Biorender.com).

Epstein–Barr nuclear antigen 1 (EBNA1) plays a crucial role in the maintenance and replication of the EBV genome within host cells. This protein is responsible for tethering the EBV episomes to the host cell chromosomes, ensuring the stable maintenance and replication of the viral genome during cell division (Figure 1) [18,25]. EBNA1 accomplishes this task through its bipartite structure: the N-terminal domain interacts with host chromatin, while the C-terminal domain binds to specific regions of the viral genome. This mechanism enables the efficient segregation of viral DNA during mitosis, facilitating the persistent infection without losing EBV genome in daughter cells [25,26].

There are multiple EBNA1 binding sites within the latent origin of plasmid replication (*OriP*), including 4 in the dyad symmetry (DS) and 18 in the family of repeats (FR) (see recent review on EBNA1-targeted inhibitors [27]). Recent cryo-electron microscopy (cryo-EM) studies have provided new insights into EBNA1 binding to DS and FR. EBNA1 binding to DS induces significant DNA bending, whereas its binding to FR results in a linear DNA structure [26].

In addition, EBNA1 also binds to the host genome and induces chromosome breakage and genome instability [28,29]. Targeting EBNA1 disrupts the tethering of EBV episomes to chromosomes, leading to the loss of the viral genome from infected cells. Since EBNA1 is consistently expressed in all EBV tumors and the EBV genome contributes to the survival and proliferation of EBV-associated tumor cells by driving oncogene expression and modulating host cellular pathways, eliminating the viral genome through EBNA1 inhibition can lead to tumor cell death.

Using the RNAi strategy, Ian et al. found that silencing EBNA1 suppresses the growth of and promotes cell cycle arrest of EBV^+^ NK/T cell lymphoma cells and HANK1 cells [30]. By using in silico virtual screening, Li et al. identified several compounds capable of inhibiting EBNA1–DNA binding in vitro and reducing EBV genome copy numbers in Raji BL cells [31]. Subsequently, Thompson et al. developed a high-throughput screening method using a homogeneous fluorescence polarization assay and identified 3 compounds (LB2, 3, and 7) that inhibit EBNA1 binding to DNA. One of the compounds, LB7, was shown to reduce EBV genome copy number in Raji BL cells [32]. Roscovitine, a cyclin-dependent kinase (CDK) inhibitor, inhibits EBNA1 phosphorylation, nuclear localization, and episome maintenance in the BJAB-derived FE1-OF cells and suppresses the growth of EBV^+^ LCLs [33].

EBNA1 forms homodimers, which are essential for its various functions, including DNA binding, replication initiation, and segregation [34,35]. Three EBNA1 dimers can form a hexameric ring that is important for plasmid maintenance [36]. Consequently, targeting EBNA1 dimerization has emerged as a promising therapeutic strategy to disrupt EBNA1 function and treat EBV-associated diseases.

EBNA1 dimerization inhibitor EiK1 and small peptide covering EBNA1 aa 560-574 blocks EBNA1’s dimerization as well as DNA binding function in vitro and in EBNA1-expressed BJAB cells [37]. Compound H31 was reported to inhibit EBNA1-*OriP* DNA binding and, therefore, reduce the growth of EBV^+^ Akata BL cells and LCLs [38]. In addition, a pyrrole-imidazole polyamide was reported to bind the EBNA1 recognition sequences as a DNA ligand to inhibit EBNA1-*OriP* binding and it suppressed the growth of EBV^+^ LCLs and limited EBV-induced B cell transformation [39]. JLP_2_ is another EBNA1 dimerization inhibitor that binds and inhibits EBNA1 in vitro and suppresses the growth of EBV^+^ NPC cells, C666-1 [40]. Subsequently, the same group developed another EBNA1-targeting probe, L_2_P_4_, based on rational design and molecular dynamics simulations [41]. L_2_P_4_ is a peptide-based inhibitor that luminesces upon binding to EBNA1 in EBV^+^ cells and selectively kills EBV^+^ NPC cells (C666-1 and NPC43) and Raji BL cells by blocking EBNA1 dimerization. Recently, a series of small molecule inhibitors were developed using structure-based design, including VK-1727 and VK-1850, that block EBNA1 DNA binding, and EBV tumor growth in vivo using xenograft models with LCLs, C666-1 NPC cells, patient-derived C15/C17 NPC cells, and EBVaGC cells (SNU719 and YCCEL1) [42,43].

Another EBNA1 inhibitor developed by the same group, VK-2019, was selected for clinical trial due to its favorable chemical and pharmacological properties [44]. Two clinical trials were initiated: An ongoing trial recruiting patients with nasopharyngeal carcinoma (NPC) and lymphoma, including post-transplant lymphoproliferative disorder (PTLD) (ClinicalTrials.gov ID: NCT04925544) and a terminated trial that focused exclusively on NPC patients (ClinicalTrials.gov ID NCT03682055). In addition to small molecule inhibitors, antibodies targeting EBNA1 DNA-binding domain have been shown to block ongoing EBV DNA replication in C666-1 NPC cells and Raji BL cells [45].

### 2.2. Targeting Viral Genome by Genome Editing

Apart from targeting EBNA1, directly disrupting the viral genome represents another way to kill virus-infected cells (Figure 1). Recent advances provide new ways to manipulate viral genome by zinc finger nucleases (ZFNs), transcription activator-like effector nucleases (TALENs), and clustered regularly interspersed short palindromic repeat (CRISPR)-associated protein 9 (Cas9) [46]. For example, Wang and Quake utilized the CRISPR/Cas9 approach to delete a portion of the EBV episome, including EBNA1, EBNA3C, and LMP1 genes. This led to reduced viral load and apoptosis of EBV^+^ BL cells [47]. van Diemen et al. used a similar method to target EBNA1 and *OriP* with double guide RNAs (gRNAs), resulting in over 95% loss of EBV genomes in Akata BL cells [48]. Subsequently, Yuen et al. tested CRISPR/Cas9 editing of EBV EBNA1, *OriP*, and W repeats in NPC cells and found that it reduces the EBV episome number by 50% [49]. Huo et al. employed CRISPR/Cas9 targeting for the LMP1 gene in NPC, which resulted in a reduction in EBV replication and decreased colony size [50].

### 2.3. Lytic Induction Therapy

Lytic induction therapy for EBV-associated cancers is a promising approach that aims to reactivate the latent virus for lytic replication [51,52,53,54,55]. Historically, chromatin-targeting drugs, such as histone deacetylase (HDAC) and DNA methyltransferase inhibitors, have been employed to induce lytic reactivation by altering the epigenetic silencing of viral lytic genes (see recent review in [56]). Recent clinical trials are evaluating the use of the HDAC inhibitor nanatinostat to induce EBV reactivation in patients with EBV^+^ lymphomas that were relapsed or refractory to prior systemic therapy. EBV reactivation leads to the expression of viral protein kinase BGLF4, a key enzyme for EBV replication that phosphorylates both viral and cellular targets [57,58,59,60,61]. BGLF4 also phosphorylated nucleoside analogs [62,63]. The activated nucleoside analogs are incorporated into viral and cellular DNA, leading to cell death. Subsequently, the lysed cells release activated analogs, which can pass through gap junctions or be transported via apoptotic vesicles to neighboring cells. This triggers cell death in adjacent cells without direct viral reactivation, a process known as bystander killing [64]. When combined with valganciclovir (vGCV), this mechanism selectively eliminates EBV-infected tumor cells, achieving an overall response rate of 40% in patients with EBV^+^ lymphoma [65] (clinicaltrials.gov IDs: NCT03397706 and NCT05011058). While these approaches have shown potential, they often lack specificity and can cause off-target effects. Indeed, nanatinostat treatment led to nausea and cytopenia as the common adverse events [65].

Emerging technologies, such as CRISPR/dCas9 activation [66,67,68,69,70,71,72,73] and transcription activator-like effector (TALE) systems [46], provide precise tools to target and activate gene expression. These approaches can be tailored to specifically reactivate EBV by targeting its immediate-early (IE) gene *ZTA/BZLF1* [74]. Recently, our group developed an approach called CRISPR/dCas9-mediated EBV reactivation (CMER) to induce EBV reactivation in cancer cells [75]. We employed an enzymatically inactive dCas9 fused with VP64 and designed guide RNAs (sgRNAs) targeting the EBV *ZTA/BZLF1* promoter. Among these, nine successfully reactivated EBV in Burkitt lymphoma cells, with CMER sgRNA-5 showing strong reactivation in multiple cancer cell types, including lymphoma, EBVaGC, and NPC. Combining CMER with GCV selectively eliminated EBV^+^ but not EBV^−^ cancer cells [75]. In another study, a ZTA/BZLF1-specific TALE system is employed to reactivate EBV in EBVaGC and NPC cells [76]. The TALE system uses nucleoside-modified mRNAs to encode a BZLF1-specific TALE-transcriptional activator. These mRNAs were then encapsulated in lipid nanoparticles (mTZ3-LNPs) for delivery to mice bearing EBV^+^ tumors xenografted with EBVaGC (SNU719) and NPC (C666-1, C17, Xeno-76) cells. The combination of mTZ3-LNP and GCV displays specific cytotoxic effects against EBV-associated tumor in vivo using these mouse models [76].

## 3. Limitations and Future Perspective

Targeting EBV episome presents significant potential for treating EBV-associated cancers. Inhibiting EBNA1 with small molecules can eliminate the EBV episome from cancer cells, while CRISPR/Cas9 editing offers a direct approach to excise the viral genome. Additionally, lytic induction therapy leverages the presence of the EBV episome to express viral protein kinase for therapeutic purposes. However, the toxicity and anti-cancer efficacy, as well as the clinical utility of EBNA1 inhibitors, are still under investigation. EBV^+^ cancer cells harbor numerous episomes, with 20–50 copies in B lymphoma cells and up to 800 copies in epithelial cancer cells [24]. As a result, completely removing all EBV genomes by CRISPR-based genome editing poses a significant challenge and may not be entirely feasible. Moreover, delivering CRISPR/Cas9 and mRNA efficiently into cancer cells also poses difficulties, as LNP-mediated mRNA delivery has shown only 15% efficiency in reactivating EBV in mouse models of EBV-associated tumors.

Several strategies could address these limitations:

PROteolysis TArgeting Chimeras (PROTACs) represents a novel approach to target previously “undruggable” proteins [77]. The PROTAC molecule acts as a bridge between the target protein and an E3 ubiquitin ligase, facilitating the formation of a ternary complex. This proximity-induced interaction triggers the ubiquitination of the target protein for ubiquitin-dependent degradation by the proteasome. Once the protein is degraded, the PROTAC is released to target another copy of the target. The catalytic nature allows a single PROTAC molecule to initiate multiple rounds of protein degradation, potentially leading to enhanced efficacy compared to traditional inhibitors [78,79,80]. Therefore, PROTAC-mediated degradation could utilize existing EBNA1 inhibitors to promote EBNA1 degradation. The PROTAC mechanism offers several advantages over conventional therapeutic approaches. By inducing protein degradation rather than mere inhibition, PROTACs may achieve more potent and sustained effects. This approach also holds promise in overcoming drug resistance mechanisms associated with traditional inhibitors due to mutations. However, the development of PROTACs faces challenges, including the optimization of physicochemical properties to ensure suitable drug-like characteristics and the enhancement of oral bioavailability [78].

In addition, new generation EBNA inhibitors with better specificity and less off-target effects could also be developed for direct inhibition or for PROTAC development. Adenoviral vectors, adeno-associated viral vectors, and lentiviral vectors -mediated CRISPR delivery might improve targeting efficiency [81]. Engineered delivery vehicles with cell-specific targeting capabilities could be developed [82,83].

Small activating RNA (saRNA) represents a novel class of therapeutic agents with unique properties that set them apart from other RNA-based therapies. Unlike small interfering RNAs (siRNAs) [84], microRNAs (miRNAs) [85], and antisense oligonucleotides [86], which typically function to silence or modulate gene expression, saRNAs are designed to upregulate specific target genes by interacting with promoter regions and recruiting RNA polymerase II [87,88,89]. This distinctive mechanism of action allows saRNAs to induce long-lasting gene expression, potentially leading to sustained therapeutic effects. In the context of EBV^+^ cancer cells, saRNAs could be adapted to target the viral IE promoter, which represents another avenue for advancing EBV-targeted therapies. The small size of saRNA may facilitate its delivery into target cells, potentially overcoming some of the delivery challenges associated with larger therapeutic molecules or gene-editing/activation systems. However, the off-target effects and stability of saRNA should be carefully examined.

Recently, the editing of the herpes simplex virus (HSV) genome by virus-specific meganucleases delivered by adeno-associated virus (AAV) vectors could eliminate over 90% of the HSV genome in mouse models [90,91]. These large and highly specific endonucleases possess the ability to recognize and cleave DNA sequences ranging from 18 to 24 base pairs in length, a characteristic that sets them apart from other gene-editing technologies [92]. The mechanism of action of meganucleases involves a series of steps, beginning with the precise recognition of their target DNA sequences. This recognition is followed by the induction of a double-strand break (DSB) at the specific site, which in turn triggers DNA repair mechanisms. These repair processes, primarily non-homologous end-joining (NHEJ) or homology-directed repair (HDR), can be harnessed to introduce desired genetic modifications, including insertions, deletions, or precise alterations when a donor DNA template is provided [93].

This strategy can also be applied to EBV by designing EBV-specific meganucleases to eliminate EBV episome in cancer cells. However, meganucleases are more difficult to develop than CRISPR/Cas9 approaches due to their larger recognition sites and the need for extensive protein engineering [92]. Additionally, the delivery mechanisms for meganucleases face similar obstacles as those encountered with CRISPR/Cas9 systems.

In conclusion, the unique presence of EBV episomes in tumor cells offers a compelling target for the development of anti-cancer therapies. This review has outlined current approaches to targeting the EBV episome, including EBNA1 inhibition, viral genome cleavage, and lytic induction through emerging strategies. Each of these methods holds the potential to disrupt cancer progression linked to EBV infection by destabilizing the episome or eradicating infected cells. However, the success of these therapeutic strategies will rely on factors such as the specific type of EBV-associated cancer, individual patient characteristics, and the potential for eliciting an immune response against EBV antigens. Future advancements in this field are likely to yield novel therapeutic avenues, offering improved strategies for treating EBV-associated malignancies.

## References

[1] Epstein M.A., Achong B.G., Barr Y.M. (1964). Virus particles in cultured lymphoblasts from burkitt’s lymphoma. Lancet.

[2] Magrath I. (2012). Epidemiology: Clues to the pathogenesis of Burkitt lymphoma. Br. J. Haematol..

[3] Grande B.M., Gerhard D.S., Jiang A., Griner N.B., Abramson J.S., Alexander T.B., Allen H., Ayers L.W., Bethony J.M., Bhatia K. (2019). Genome-wide discovery of somatic coding and noncoding mutations in pediatric endemic and sporadic Burkitt lymphoma. Blood.

[4] Balfour H.H., Sifakis F., Sliman J.A., Knight J.A., Schmeling D.O., Thomas W. (2013). Age-specific prevalence of Epstein-Barr virus infection among individuals aged 6-19 years in the United States and factors affecting its acquisition. J. Infect. Dis..

[5] Hjalgrim H., Askling J., Rostgaard K., Hamilton-Dutoit S., Frisch M., Zhang J.S., Madsen M., Rosdahl N., Konradsen H.B., Storm H.H. (2003). Characteristics of Hodgkin’s lymphoma after infectious mononucleosis. N. Engl. J. Med..

[6] Xu M., Feng R., Liu Z., Zhou X., Chen Y., Cao Y., Valeri L., Li Z., Liu Z., Cao S.M. (2024). Host genetic variants, Epstein-Barr virus subtypes, and the risk of nasopharyngeal carcinoma: Assessment of interaction and mediation. Cell Genom..

[7] Zeng Y., Luo C.L., Lin G.W., Li F., Bai X., Ko J.M., Xiong L., Liu Y., He S., Jiang J.X. (2025). Whole-exome sequencing association study reveals genetic effects on tumor microenvironment components in nasopharyngeal carcinoma. J. Clin. Investig..

[8] Young L.S., Dawson C.W. (2014). Epstein-Barr virus and nasopharyngeal carcinoma. Chin. J. Cancer.

[9] Burke A.P., Yen T.S., Shekitka K.M., Sobin L.H. (1990). Lymphoepithelial carcinoma of the stomach with Epstein-Barr virus demonstrated by polymerase chain reaction. Mod. Pathol..

[10] Stanland L.J., Luftig M.A. (2020). The Role of EBV-Induced Hypermethylation in Gastric Cancer Tumorigenesis. Viruses.

[11] Kojima Y., Hamada M., Naruse A., Goto K., Khine H.T., Arai H., Akutsu Y., Satou A., Nakaguro M., Kato S. (2024). The landscape of 142 Epstein-Barr viral whole genomes in gastric cancer. J. Gastroenterol..

[12] Young L.S., Yap L.F., Murray P.G. (2016). Epstein-Barr virus: More than 50 years old and still providing surprises. Nat. Rev. Cancer.

[13] Damania B., Kenney S.C., Raab-Traub N. (2022). Epstein-Barr virus: Biology and clinical disease. Cell.

[14] Bjornevik K., Münz C., Cohen J.I., Ascherio A. (2023). Epstein-Barr virus as a leading cause of multiple sclerosis: Mechanisms and implications. Nat. Rev. Neurol..

[15] Soldan S.S., Lieberman P.M. (2023). Epstein-Barr virus and multiple sclerosis. Nat. Rev. Microbiol..

[16] Lanz T.V., Brewer R.C., Ho P.P., Moon J.S., Jude K.M., Fernandez D., Fernandes R.A., Gomez A.M., Nadj G.S., Bartley C.M. (2022). Clonally expanded B cells in multiple sclerosis bind EBV EBNA1 and GlialCAM. Nature.

[17] Bjornevik K., Cortese M., Healy B.C., Kuhle J., Mina M.J., Leng Y., Elledge S.J., Niebuhr D.W., Scher A.I., Munger K.L. (2022). Longitudinal analysis reveals high prevalence of Epstein-Barr virus associated with multiple sclerosis. Science.

[18] Lieberman P.M. (2013). Keeping it quiet: Chromatin control of gammaherpesvirus latency. Nat. Rev. Microbiol..

[19] Shimizu N., Yoshiyama H., Takada K. (1996). Clonal propagation of Epstein-Barr virus (EBV) recombinants in EBV-negative Akata cells. J. Virol..

[20] Nanbo A., Katano H., Kataoka M., Hoshina S., Sekizuka T., Kuroda M., Ohba Y. (2018). Infection of Epstein–Barr Virus in Type III Latency Modulates Biogenesis of Exosomes and the Expression Profile of Exosomal miRNAs in the Burkitt Lymphoma Mutu Cell Lines. Cancers.

[21] Pan Y.R., Fang C.Y., Chang Y.S., Chang H.Y. (2005). Analysis of Epstein-Barr virus gene expression upon phorbol ester and hydroxyurea treatment by real-time quantitative PCR. Arch. Virol..

[22] Zuo L., Yu H., Liu L., Tang Y., Wu H., Yang J., Zhu M., Du S., Zhao L., Cao L. (2015). The copy number of Epstein-Barr virus latent genome correlates with the oncogenicity by the activation level of LMP1 and NF-κB. Oncotarget.

[23] Houldcroft C.J., Petrova V., Liu J.Z., Frampton D., Anderson C.A., Gall A., Kellam P. (2014). Host genetic variants and gene expression patterns associated with Epstein-Barr virus copy number in lymphoblastoid cell lines. PLoS ONE.

[24] Kim D.N., Seo M.K., Choi H., Kim S.Y., Shin H.J., Yoon A.R., Tao Q., Rha S.Y., Lee S.K. (2013). Characterization of naturally Epstein-Barr virus-infected gastric carcinoma cell line YCCEL1. J. Gen. Virol..

[25] Dheekollu J., Wiedmer A., Ayyanathan K., Deakyne J.S., Messick T.E., Lieberman P.M. (2021). Cell-cycle-dependent EBNA1-DNA crosslinking promotes replication termination at oriP and viral episome maintenance. Cell.

[26] Mei Y., Messick T.E., Dheekollu J., Kim H.J., Molugu S., Muñoz L.J.C., Moiskeenkova-Bell V., Murakami K., Lieberman P.M. (2022). Cryo-EM Structure and Functional Studies of EBNA1 Binding to the Family of Repeats and Dyad Symmetry Elements of Epstein-Barr Virus oriP. J. Virol..

[27] Jiang L., Xie C., Lung H.L., Lo K.W., Law G.L., Mak N.K., Wong K.L. (2018). EBNA1-targeted inhibitors: Novel approaches for the treatment of Epstein-Barr virus-associated cancers. Theranostics.

[28] Li J.S.Z., Abbasi A., Kim D.H., Lippman S.M., Alexandrov L.B., Cleveland D.W. (2023). Chromosomal fragile site breakage by EBV-encoded EBNA1 at clustered repeats. Nature.

[29] Lu F., Wikramasinghe P., Norseen J., Tsai K., Wang P., Showe L., Davuluri R.V., Lieberman P.M. (2010). Genome-wide analysis of host-chromosome binding sites for Epstein-Barr Virus Nuclear Antigen 1 (EBNA1). Virol. J..

[30] Ian M.X., Lan S.Z., Cheng Z.F., Dan H., Qiong L.H. (2008). Suppression of EBNA1 expression inhibits growth of EBV-positive NK/T cell lymphoma cells. Cancer Biol. Ther..

[31] Li N., Thompson S., Schultz D.C., Zhu W., Jiang H., Luo C., Lieberman P.M. (2010). Discovery of selective inhibitors against EBNA1 via high throughput in silico virtual screening. PLoS ONE.

[32] Thompson S., Messick T., Schultz D.C., Reichman M., Lieberman P.M. (2010). Development of a high-throughput screen for inhibitors of Epstein-Barr virus EBNA1. J. Biomol. Screen..

[33] Kang M.S., Lee E.K., Soni V., Lewis T.A., Koehler A.N., Srinivasan V., Kieff E. (2011). Roscovitine inhibits EBNA1 serine 393 phosphorylation, nuclear localization, transcription, and episome maintenance. J. Virol..

[34] Bochkarev A., Barwell J.A., Pfuetzner R.A., Furey W., Edwards A.M., Frappier L. (1995). Crystal structure of the DNA-binding domain of the Epstein-Barr virus origin-binding protein EBNA 1. Cell.

[35] Cruickshank J., Shire K., Davidson A.R., Edwards A.M., Frappier L. (2000). Two domains of the epstein-barr virus origin DNA-binding protein, EBNA1, orchestrate sequence-specific DNA binding. J. Biol. Chem..

[36] Deakyne J.S., Malecka K.A., Messick T.E., Lieberman P.M. (2017). Structural and Functional Basis for an EBNA1 Hexameric Ring in Epstein-Barr Virus Episome Maintenance. J. Virol..

[37] Kim S.Y., Song K.A., Kieff E., Kang M.S. (2012). Small molecule and peptide-mediated inhibition of Epstein-Barr virus nuclear antigen 1 dimerization. Biochem. Biophys. Res. Commun..

[38] Lee E.K., Kim S.Y., Noh K.W., Joo E.H., Zhao B., Kieff E., Kang M.S. (2014). Small molecule inhibition of Epstein-Barr virus nuclear antigen-1 DNA binding activity interferes with replication and persistence of the viral genome. Antiviral Res..

[39] Yasuda A., Noguchi K., Minoshima M., Kashiwazaki G., Kanda T., Katayama K., Mitsuhashi J., Bando T., Sugiyama H., Sugimoto Y. (2011). DNA ligand designed to antagonize EBNA1 represses Epstein-Barr virus-induced immortalization. Cancer Sci..

[40] Jiang L., Lui Y.L., Li H., Chan C.F., Lan R., Chan W.L., Lau T.C., Tsao G.S., Mak N.K., Wong K.L. (2014). EBNA1-specific luminescent small molecules for the imaging and inhibition of latent EBV-infected tumor cells. Chem. Commun..

[41] Jiang L., Lan R., Huang T., Chan C.-F., Li H., Lear S., Zong J., Wong W.-Y., Muk-Lan Lee M., Dow Chan B. (2017). EBNA1-targeted probe for the imaging and growth inhibition of tumours associated with the Epstein–Barr virus. Nat. Biomed. Eng..

[42] Messick T.E., Smith G.R., Soldan S.S., McDonnell M.E., Deakyne J.S., Malecka K.A., Tolvinski L., van den Heuvel A.P.J., Gu B.W., Cassel J.A. (2019). Structure-based design of small-molecule inhibitors of EBNA1 DNA binding blocks Epstein-Barr virus latent infection and tumor growth. Sci. Transl. Med..

[43] Soldan S.S., Anderson E.M., Frase D.M., Zhang Y., Caruso L.B., Wang Y., Deakyne J.S., Gewurz B.E., Tempera I., Lieberman P.M. (2021). EBNA1 inhibitors have potent and selective antitumor activity in xenograft models of Epstein-Barr virus-associated gastric cancer. Gastric Cancer.

[44] Davis M.T., Anders N.M., Colevas A.D., Messick T.E., Rudek M.A. (2024). Validation of a robust and rapid liquid chromatography tandem mass spectrometric method for the quantitative analysis of VK-2019, a selective EBNA1 inhibitor. Biomed. Chromatogr..

[45] Han Y., Wu F., Zhang Y., Liu J., Wu Y., Wang Y., Jiang X., Chen X., Xu W. (2024). Structure-based design of antibodies targeting the EBNA1 DNA-binding domain to block Epstein-Barr virus latent infection and tumor growth. MedComm.

[46] Li H., Yang Y., Hong W., Huang M., Wu M., Zhao X. (2020). Applications of genome editing technology in the targeted therapy of human diseases: Mechanisms, advances and prospects. Signal Transduct. Target. Ther..

[47] Wang J., Quake S.R. (2014). RNA-guided endonuclease provides a therapeutic strategy to cure latent herpesviridae infection. Proc. Natl. Acad. Sci. USA.

[48] van Diemen F.R., Kruse E.M., Hooykaas M.J., Bruggeling C.E., Schürch A.C., van Ham P.M., Imhof S.M., Nijhuis M., Wiertz E.J., Lebbink R.J. (2016). CRISPR/Cas9-Mediated Genome Editing of Herpesviruses Limits Productive and Latent Infections. PLoS Pathog..

[49] Yuen K.S., Wang Z.M., Wong N.M., Zhang Z.Q., Cheng T.F., Lui W.Y., Chan C.P., Jin D.Y. (2018). Suppression of Epstein-Barr virus DNA load in latently infected nasopharyngeal carcinoma cells by CRISPR/Cas9. Virus Res..

[50] Huo H., Hu G. (2019). CRISPR/Cas9-mediated LMP1 knockout inhibits Epstein-Barr virus infection and nasopharyngeal carcinoma cell growth. Infect. Agent. Cancer.

[51] Feng W.H., Israel B., Raab-Traub N., Busson P., Kenney S.C. (2002). Chemotherapy induces lytic EBV replication and confers ganciclovir susceptibility to EBV-positive epithelial cell tumors. Cancer Res..

[52] Feng W.H., Westphal E., Mauser A., Raab-Traub N., Gulley M.L., Busson P., Kenney S.C. (2002). Use of adenovirus vectors expressing Epstein-Barr virus (EBV) immediate-early protein BZLF1 or BRLF1 to treat EBV-positive tumors. J. Virol..

[53] Fu D.X., Tanhehco Y., Chen J., Foss C.A., Fox J.J., Chong J.M., Hobbs R.F., Fukayama M., Sgouros G., Kowalski J. (2008). Bortezomib-induced enzyme-targeted radiation therapy in herpesvirus-associated tumors. Nat. Med..

[54] Westphal E.M., Blackstock W., Feng W., Israel B., Kenney S.C. (2000). Activation of lytic Epstein-Barr virus (EBV) infection by radiation and sodium butyrate in vitro and in vivo: A potential method for treating EBV-positive malignancies. Cancer Res..

[55] Westphal E.M., Mauser A., Swenson J., Davis M.G., Talarico C.L., Kenney S.C. (1999). Induction of lytic Epstein-Barr virus (EBV) infection in EBV-associated malignancies using adenovirus vectors in vitro and in vivo. Cancer Res..

[56] Yiu S.P.T., Dorothea M., Hui K.F., Chiang A.K.S. (2020). Lytic Induction Therapy against Epstein-Barr Virus-Associated Malignancies: Past, Present, and Future. Cancers.

[57] Li R., Liao G., Nirujogi R.S., Pinto S.M., Shaw P.G., Huang T.C., Wan J., Qian J., Gowda H., Wu X. (2015). Phosphoproteomic Profiling Reveals Epstein-Barr Virus Protein Kinase Integration of DNA Damage Response and Mitotic Signaling. PLoS Pathog..

[58] Zhang K., Lv D.W., Li R. (2019). Conserved Herpesvirus Protein Kinases Target SAMHD1 to Facilitate Virus Replication. Cell Rep..

[59] Wang J.T., Yang P.W., Lee C.P., Han C.H., Tsai C.H., Chen M.R. (2005). Detection of Epstein-Barr virus BGLF4 protein kinase in virus replication compartments and virus particles. J. Gen. Virol..

[60] Li R., Zhu J., Xie Z., Liao G., Liu J., Chen M.R., Hu S., Woodard C., Lin J., Taverna S.D. (2011). Conserved herpesvirus kinases target the DNA damage response pathway and TIP60 histone acetyltransferase to promote virus replication. Cell Host Microbe.

[61] Zhu J., Liao G., Shan L., Zhang J., Chen M.R., Hayward G.S., Hayward S.D., Desai P., Zhu H. (2009). Protein array identification of substrates of the Epstein-Barr virus protein kinase BGLF4. J. Virol..

[62] Meng Q., Hagemeier S.R., Fingeroth J.D., Gershburg E., Pagano J.S., Kenney S.C. (2010). The Epstein-Barr virus (EBV)-encoded protein kinase, EBV-PK, but not the thymidine kinase (EBV-TK), is required for ganciclovir and acyclovir inhibition of lytic viral production. J. Virol..

[63] Li R., Hayward S.D. (2013). Potential of protein kinase inhibitors for treating herpesvirus-associated disease. Trends Microbiol..

[64] Colombo B.M., Benedetti S., Ottolenghi S., Mora M., Pollo B., Poli G., Finocchiaro G. (1995). The “bystander effect”: Association of U-87 cell death with ganciclovir-mediated apoptosis of nearby cells and lack of effect in athymic mice. Hum. Gene Ther..

[65] Haverkos B., Alpdogan O., Baiocchi R., Brammer J.E., Feldman T.A., Capra M., Brem E.A., Nair S., Scheinberg P., Pereira J. (2023). Targeted therapy with nanatinostat and valganciclovir in recurrent EBV-positive lymphoid malignancies: A phase 1b/2 study. Blood Adv..

[66] Cong L., Ran F.A., Cox D., Lin S., Barretto R., Habib N., Hsu P.D., Wu X., Jiang W., Marraffini L.A. (2013). Multiplex genome engineering using CRISPR/Cas systems. Science.

[67] Gebre M., Nomburg J.L., Gewurz B.E. (2018). CRISPR-Cas9 Genetic Analysis of Virus-Host Interactions. Viruses.

[68] Mali P., Yang L., Esvelt K.M., Aach J., Guell M., DiCarlo J.E., Norville J.E., Church G.M. (2013). RNA-guided human genome engineering via Cas9. Science.

[69] Wang L.W., Trudeau S.J., Wang C., Gerdt C., Jiang S., Zhao B., Gewurz B.E. (2018). Modulating Gene Expression in Epstein-Barr Virus (EBV)-Positive B Cell Lines with CRISPRa and CRISPRi. Curr. Protoc. Mol. Biol..

[70] Cheng A.W., Wang H., Yang H., Shi L., Katz Y., Theunissen T.W., Rangarajan S., Shivalila C.S., Dadon D.B., Jaenisch R. (2013). Multiplexed activation of endogenous genes by CRISPR-on, an RNA-guided transcriptional activator system. Cell Res..

[71] Konermann S., Brigham M.D., Trevino A.E., Joung J., Abudayyeh O.O., Barcena C., Hsu P.D., Habib N., Gootenberg J.S., Nishimasu H. (2015). Genome-scale transcriptional activation by an engineered CRISPR-Cas9 complex. Nature.

[72] Maeder M.L., Linder S.J., Cascio V.M., Fu Y., Ho Q.H., Joung J.K. (2013). CRISPR RNA-guided activation of endogenous human genes. Nat. Methods.

[73] Perez-Pinera P., Kocak D.D., Vockley C.M., Adler A.F., Kabadi A.M., Polstein L.R., Thakore P.I., Glass K.A., Ousterout D.G., Leong K.W. (2013). RNA-guided gene activation by CRISPR-Cas9-based transcription factors. Nat. Methods.

[74] Kenney S.C., Mertz J.E. (2014). Regulation of the latent-lytic switch in Epstein-Barr virus. Semin. Cancer Biol..

[75] Sugiokto F.G., Li R. (2024). Targeted eradication of EBV-positive cancer cells by CRISPR/dCas9-mediated EBV reactivation in combination with ganciclovir. mBio.

[76] Wu M., Hau P.M., Li L., Tsang C.M., Yang Y., Taghbalout A., Chung G.T., Hui S.Y., Tang W.C., Jillette N. (2024). Synthetic BZLF1-targeted transcriptional activator for efficient lytic induction therapy against EBV-associated epithelial cancers. Nat. Commun..

[77] Bond M.J., Crews C.M. (2021). Proteolysis targeting chimeras (PROTACs) come of age: Entering the third decade of targeted protein degradation. RSC Chem. Biol..

[78] Békés M., Langley D.R., Crews C.M. (2022). PROTAC targeted protein degraders: The past is prologue. Nat. Rev. Drug Discov..

[79] Sakamoto K.M., Kim K.B., Kumagai A., Mercurio F., Crews C.M., Deshaies R.J. (2001). Protacs: Chimeric molecules that target proteins to the Skp1-Cullin-F box complex for ubiquitination and degradation. Proc. Natl. Acad. Sci. USA.

[80] Lu J., Qian Y., Altieri M., Dong H., Wang J., Raina K., Hines J., Winkler J.D., Crew A.P., Coleman K. (2015). Hijacking the E3 Ubiquitin Ligase Cereblon to Efficiently Target BRD4. Chem. Biol..

[81] Asmamaw Mengstie M. (2022). Viral Vectors for the in Vivo Delivery of CRISPR Components: Advances and Challenges. Front. Bioeng. Biotechnol..

[82] Hamilton J.R., Chen E., Perez B.S., Sandoval Espinoza C.R., Kang M.H., Trinidad M., Ngo W., Doudna J.A. (2024). In vivo human T cell engineering with enveloped delivery vehicles. Nat. Biotechnol..

[83] Hamilton J.R., Tsuchida C.A., Nguyen D.N., Shy B.R., McGarrigle E.R., Sandoval Espinoza C.R., Carr D., Blaeschke F., Marson A., Doudna J.A. (2021). Targeted delivery of CRISPR-Cas9 and transgenes enables complex immune cell engineering. Cell Rep..

[84] Fire A., Xu S., Montgomery M.K., Kostas S.A., Driver S.E., Mello C.C. (1998). Potent and specific genetic interference by double-stranded RNA in Caenorhabditis elegans. Nature.

[85] Lee R.C., Feinbaum R.L., Ambros V. (1993). The C. elegans heterochronic gene lin-4 encodes small RNAs with antisense complementarity to lin-14. Cell.

[86] Stephenson M.L., Zamecnik P.C. (1978). Inhibition of Rous sarcoma viral RNA translation by a specific oligodeoxyribonucleotide. Proc. Natl. Acad. Sci. USA.

[87] Portnoy V., Lin S.H., Li K.H., Burlingame A., Hu Z.H., Li H., Li L.C. (2016). saRNA-guided Ago2 targets the RITA complex to promoters to stimulate transcription. Cell Res..

[88] Jiao A.L., Slack F.J. (2014). RNA-mediated gene activation. Epigenetics.

[89] Li L.C., Okino S.T., Zhao H., Pookot D., Place R.F., Urakami S., Enokida H., Dahiya R. (2006). Small dsRNAs induce transcriptional activation in human cells. Proc. Natl. Acad. Sci. USA.

[90] Aubert M., Haick A.K., Strongin D.E., Klouser L.M., Loprieno M.A., Stensland L., Santo T.K., Huang M.L., Hyrien O., Stone D. (2024). Gene editing for latent herpes simplex virus infection reduces viral load and shedding in vivo. Nat. Commun..

[91] Aubert M., Strongin D.E., Roychoudhury P., Loprieno M.A., Haick A.K., Klouser L.M., Stensland L., Huang M.L., Makhsous N., Tait A. (2020). Gene editing and elimination of latent herpes simplex virus in vivo. Nat. Commun..

[92] Takeuchi R., Choi M., Stoddard B.L. (2015). Engineering of customized meganucleases via in vitro compartmentalization and in cellulo optimization. Methods Mol. Biol..

[93] Silva G., Poirot L., Galetto R., Smith J., Montoya G., Duchateau P., Pâques F. (2011). Meganucleases and other tools for targeted genome engineering: Perspectives and challenges for gene therapy. Curr. Gene Ther..

